# Supplementary material for: Developing a recombinase-aided amplification method combined with a lateral flow dipstick assay for rapid triplex detection of bovine coronavirus, infectious bovine rhinotracheitis virus, and bovine viral diarrhea virus
Source: Microbiol Spectr. 2025 Nov 26;14(1):e01628-25. doi: 10.1128/spectrum.01628-25 (PMC12772406; doi:10.1128/spectrum.01628-25)
Supplement: Supplemental material — Fig. S1 and S2; Supplemental table legends. [file spectrum.01628-25-s0001.pdf]

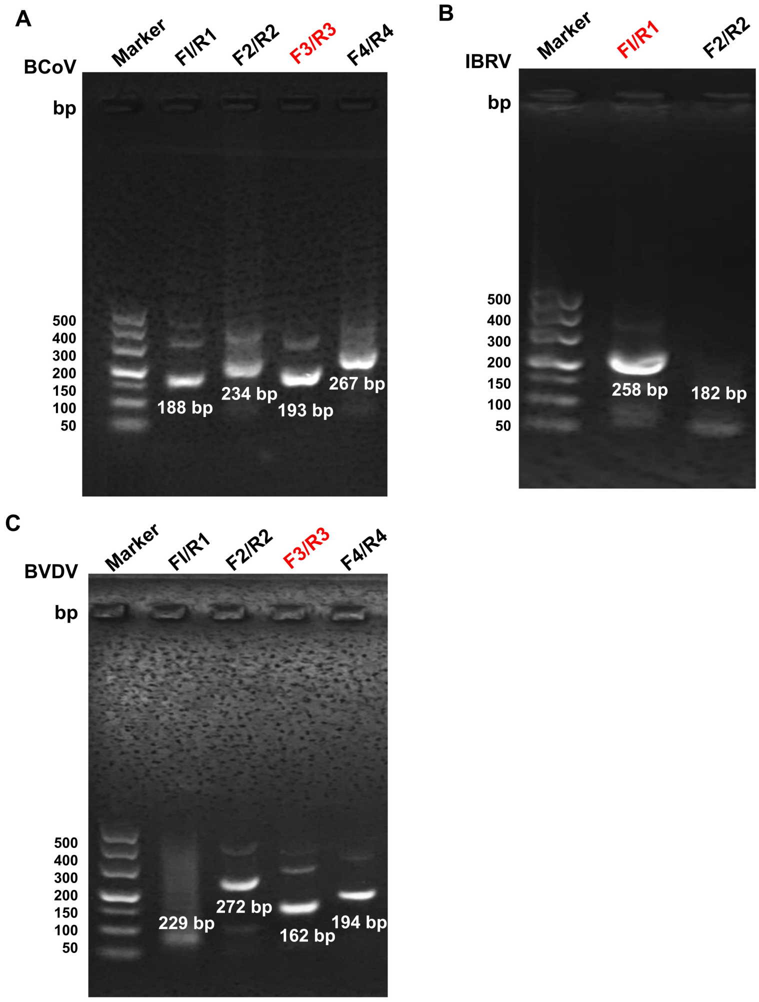

Figure S1. Screening of primary primers for the RAA assay. (A) RAA amplification of BCoV. Lane M is a 50-500 bp ladder marker, while lanes 2-4 are the results of RAA amplification of BCoV via the primers RAA-F1/R1, RAA-F2/R2, RAA-F3/R3 and RAA-F4/R4. **(B) RAA amplification of IBRV.** Lane M was a 50-500 bp ladder marker, whereas lanes 2 and 3 were the results of RAA amplification of IBRV via the primers RAA-F1/R1 and RAA-F2/R2. **(C) RAA amplification of BVDV.** Lane M is a 50-500 bp ladder marker, while lanes 2-4 are the results of RAA amplification of BVDV via the primers RAA-F1/R1, RAA-F2/R2, RAA-F3/R3, and RAA-F4/R4.

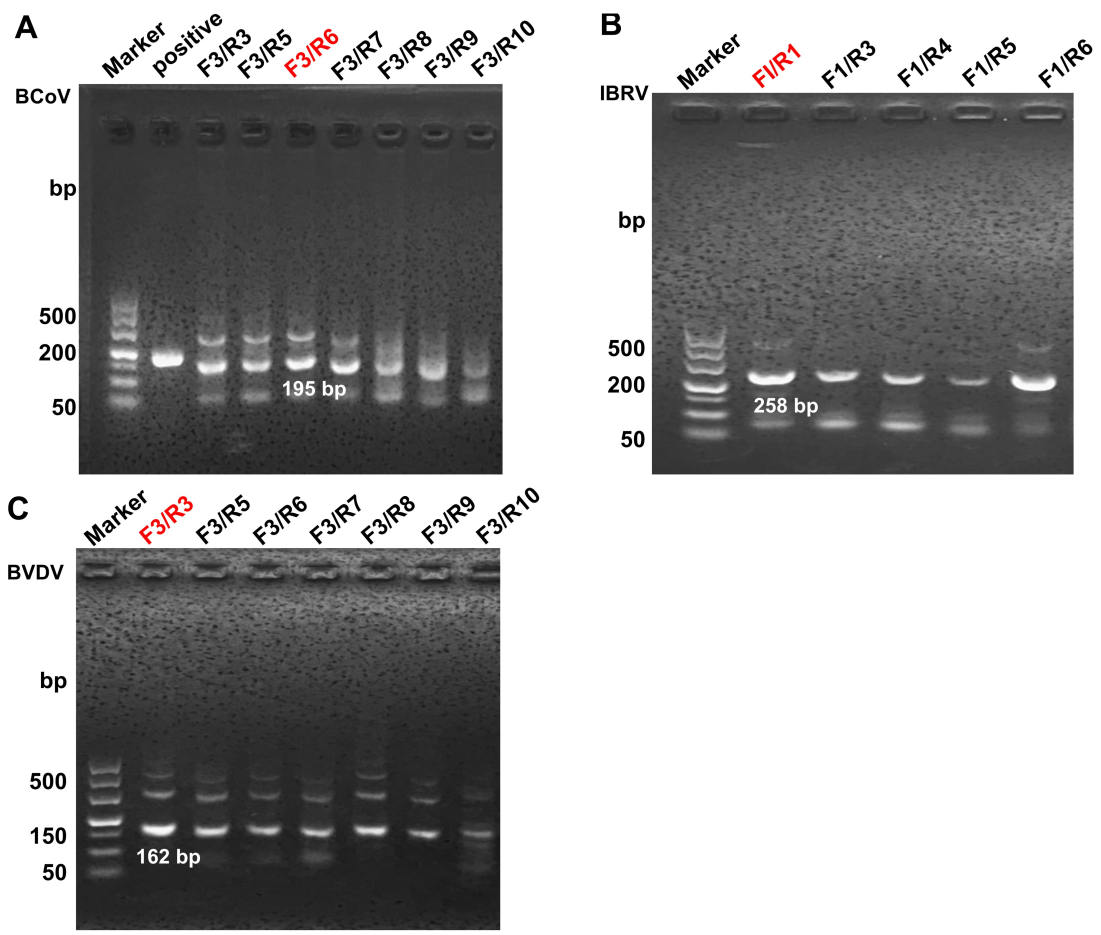

Figure S2. Screening of secondary primers for the RAA assay. (A) Fixed upstream primers were used to select downstream primers. Lane M was a 50-500 bp ladder marker, Lane 2 was a positive control, and Lanes 3-9 were the results of RAA amplification of BCoV via the primers RAA-F3/R3, RAA-F3/R5, RAA-F3/R6, RAA-F3/R7, RAA-F3/R8, RAA-F3/R9 and RAA-F3/R10. **(B) Fixed upstream primers were used to select downstream primers.** Lane M is a 50-500 bp ladder marker, while lanes 2-6 are the results of RAA amplification of IBRV via the primers RAA-F1/R1, RAA-F1/R3, RAA-F1/R4, RAA-F1/R5 and RAA-F1/R6. **(C) Fixed upstream primers were used to select downstream primers.** Lane M is a 50-500 bp ladder marker, while lanes 2-8 are the results of RAA amplification of BVDV via the primers RAA-F3/R3, RAA-F3/R5, RAA-F3/R6, RAA-F3/R7, RAA-F3/R8, RAA-F3/R9 and RAA-F3/R10.

Supplemental material

Figure S1. Screening of primary primers of the RAA assay.

Figure S2. Screening of secondary primers of the RAA assay.

Table S1. RAA assay primers of BCoV, IBRV and BVDV.

Table S2. Primers of PCR and RT-qPCR and probes.

Table S3. The optimum primers and probes of RAA-LFD.

Table S4. The sample test statistics.
